# Detection and Identification of Tassel States at Different Maize Tasseling Stages Using UAV Imagery and Deep Learning

**DOI:** 10.34133/plantphenomics.0188

**Published:** 2024-06-26

**Authors:** Jianjun Du, Jinrui Li, Jiangchuan Fan, Shenghao Gu, Xinyu Guo, Chunjiang Zhao

**Affiliations:** ^1^ Information Technology Research Center, Beijing Academy of Agriculture and Forestry Sciences, Beijing 100097, China.; ^2^ Beijing Key Lab of Digital Plants, National Engineering Research Center for Information Technology in Agriculture, Beijing 100097, China.; ^3^ College of Information Engineering, Northwest A&F University, Yangling, Shanxi 712100 China.

## Abstract

The tassel state in maize hybridization fields not only reflects the growth stage of the maize but also reflects the performance of the detasseling operation. Existing tassel detection models are primarily used to identify mature tassels with obvious features, making it difficult to accurately identify small tassels or detasseled plants. This study presents a novel approach that utilizes unmanned aerial vehicles (UAVs) and deep learning techniques to accurately identify and assess tassel states, before and after manually detasseling in maize hybridization fields. The proposed method suggests that a specific tassel annotation and data augmentation strategy is valuable for substantial enhancing the quality of the tassel training data. This study also evaluates mainstream object detection models and proposes a series of highly accurate tassel detection models based on tassel categories with strong data adaptability. In addition, a strategy for blocking large UAV images, as well as improving tassel detection accuracy, is proposed to balance UAV image acquisition and computational cost. The experimental results demonstrate that the proposed method can accurately identify and classify tassels at various stages of detasseling. The tassel detection model optimized with the enhanced data achieves an average precision of 94.5% across all categories. An optimal model combination that uses blocking strategies for different development stages can improve the tassel detection accuracy to 98%. This could be useful in addressing the issue of missed tassel detections in maize hybridization fields. The data annotation strategy and image blocking strategy may also have broad applications in object detection and recognition in other agricultural scenarios.

## Introduction

Maize is one of the most important crops in China. Monitoring the tasseling stage is essential for maize breeding operations. In recent years, unmanned aerial vehicles (UAVs) have been widely used for maize planting, management, and harvesting due to their low cost, high efficiency, and flexibility and thus have an irreplaceable advantage in maize growth monitoring. Recent advances in remote sensing technologies and data processing have made UAVs valuable tools for obtaining detailed data on plant diseases [1], predicting maize grain yield [2], and counting maize plants [3]. However, these image-based UAV applications have generated massive amounts of image data, which presents both opportunities and challenges. The use of powerful deep learning techniques to automatically process images and analyze phenotypic traits remains a critical but unsolved challenge that warrants further research in this area. Overcoming this barrier will unlock the full potential of UAV-based crop growth monitoring and accelerated breeding through robust image phenotyping.

Currently, most maize tassel detection methods are based on deep learning frameworks using convolutional neural networks (CNNs) as the core inference engine [3–6]. Traditional image processing techniques are usually used to complement these CNN models for pre- and postprocessing operations [1,7,8]. Given the challenges of data acquisition and labeling in agricultural scenes, traditional image processing techniques, such as *K*-means clustering with adaptive thresholding, can be effective only in specific scenarios or when the characteristics of mature tassels are highly distinct [4] and be used to detect maize tassels. In recent years, many studies on tassel detection based on UAV imagery have emerged. These studies, based on existing detection network structures, typically improve models by replacing feature detection heads, integrating attention mechanisms, reducing model complexity, etc., to achieve better relative performances than the benchmark models. For example, a YOLOv5 (You Only Look Once v5)-based tassel model was developed to detect tassels in UAV-based RGB (red–green–blue) images and achieved a mean average precision (mAP) of 44.7%, which is better than that of well-known object detection approaches, such as FCOS, RetinaNet, and YOLOv5[9]. Furthermore, an enhanced RetinaNet with an attention mechanism has been proposed to improve the detection of small objects, achieving a higher precision (0.9717) than Faster R-CNN, YOLOX, and SSD [10]. Another recent study focused on improving the YOLOv7 model by adding a global attention mechanism, adopting a GSConv convolution and a VoVGSCSP module in the neck part, and changing the loss function to an SIoU loss function; the mAP@0.5 of Tassel-YOLO reached 96.14% [11]. In addition, YOLOX was extended by embedding an attention mechanism to implement critical feature extractions and the suppression of noise caused by interfering factors (occlusions, overlaps, etc.), and the improved model had a slight increase in detection accuracy [12]. A lightweight neural network named TasselLFANet has been proposed to accurately and efficiently detect and count tassels in high-spatiotemporal-resolution image sequences. The network uses cross-stage fusion strategies, multireceptive field feature expressions, and innovative visual channel attention modules to enhance the feature learning ability. Its statistical performance is better than that of TasselNetV3-Seg†, with an *F*_1_ value of 94.4% [13]. CenterNet was also improved using point annotation and task-aware spatial disentanglement techniques for tassel detection. This approach has been shown to be more robust than Tasselnetv2+ [14] since it is sensitive to the number of tassels in the image [4]. Some older detection models have been used to detect maize tassels. For example, Faster R-CNN with ResNet and VGGNet was evaluated in [5] and compared with TasselNet [15]. Before improving the tassel detection models based on CNNs, a comprehensive benchmark of the state-of-the-art tassel detection and object counting methods was presented [6]. The benchmarks included Faster R-CNN, YOLOv3, FaceBoxes, RetinaNet, and TasselNet.

The above progress in the tassel detection models still depends on the integration of different detection model architectures from both academia and industry. This is followed by model improvements in areas such as feature extractions [12,16], attention mechanisms [9,10,17], and model lightweighting [13] to facilitate training or tuning on customized datasets. The effectiveness of the enhanced data optimization strategies for these models has not been thoroughly tested or evaluated, as there are few experimental reports available. When defining the semantics of tassel data, only mature tassels with distinct features are annotated and identified as a single category. However, this approach does not consider the dynamic growth characteristics of real tassels across the developmental stages or the significant differences between tassel features at each stage. In addition, current benchmarks for model accuracy and performance may not be fair. The evaluations mainly use small test images, without considering the potential for severe perspective distortions of tassels at different locations in large UAV images. This distortion could have a significant impact on the tassel detection capabilities.

This study aims to address the need for accurate detection and identification of maize tassel growth stages in maize hybridization fields [18]. To achieve this goal, a suite of annotated datasets capturing the dynamic developmental stages of maize tassels is created. The data annotation and augmentation strategies used to represent multistage tassel features are evaluated, and detection models are benchmarked on these datasets to select optimal network architectures and appropriate detection models. Quantitative tests based on blocking patterns are performed on large-scale UAV imagery to provide recommendations for advancing the development of maize tassel detection and facilitating real-world deployment.

## Materials and Methods

### Image acquisition

The data collection experiments were conducted from 30 June to 4 July 2022 in Tacheng City, Xinjiang Region, China to collect data on maize hybridization. Confidential commercial maize varieties were planted in different spatial arrangements in 5 fields, with both female and male plants. Before and after manual removal of the tassels, a DJI Matrice 300 RTK was used to capture top-view images of the maize canopy in the field. To reduce the effects of variable illumination and plant movement on the quality of the images, low-altitude flights were deliberately conducted under overcast skies and windless conditions following a prescribed route plan. A UAV captured high-quality RGB images using a 45-megapixel full-frame camera (Zenmuse P1). The individual images were captured at a resolution of 8,192 × 5,460 pixels, with a ground sampling distance (GSD) of 0.25 cm/pixel. The ground sampling distance depends only on the flight altitude for a fixed focal length. Hundreds of images were collected per mission during continuous flight sessions of approximately 30 min. A subset of these images was selected to construct training and test datasets for the tassel detection models.

### Data processing strategy

**Fig. 1.**
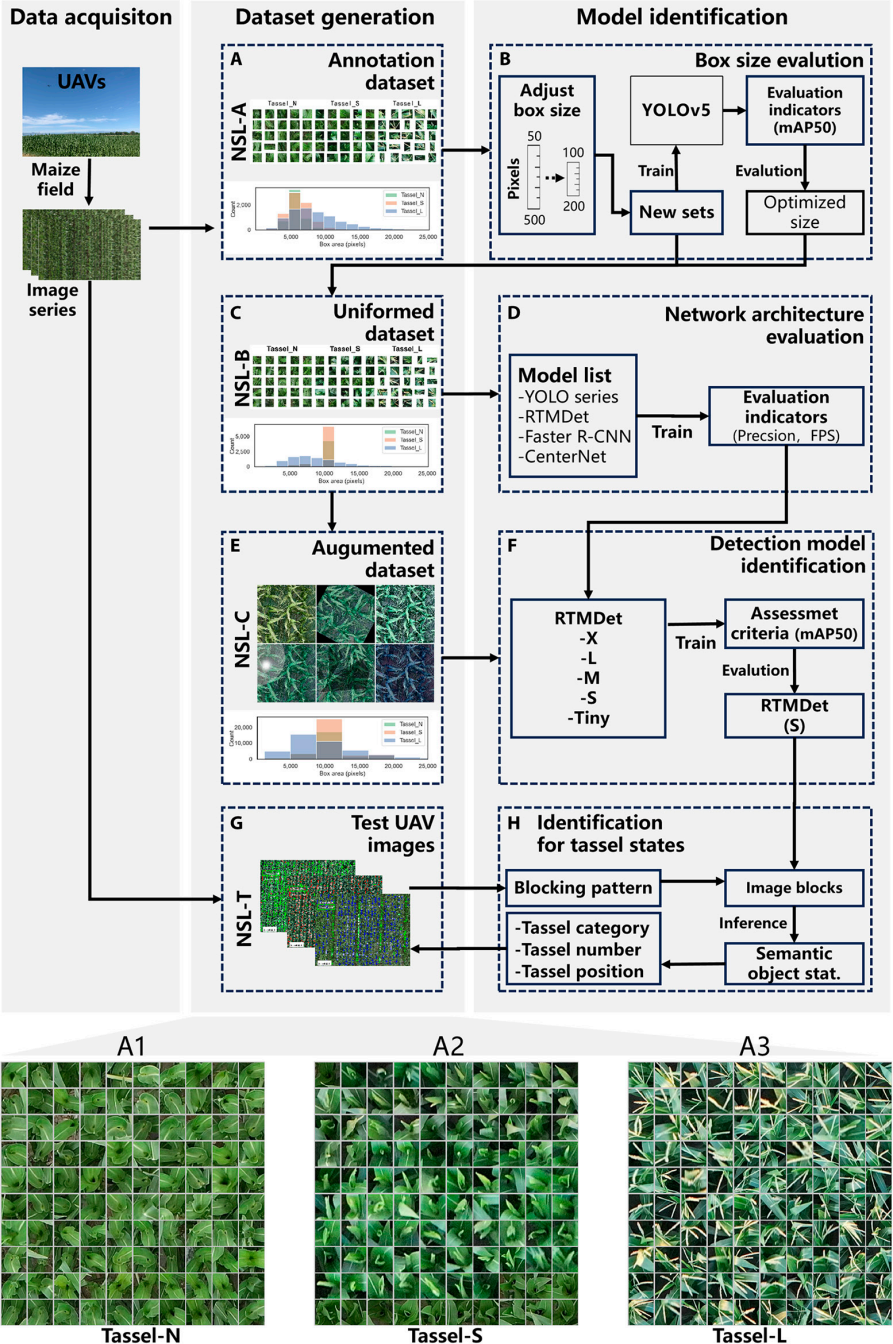
Flowchart for the detection and identification of maize tassel states in maize hybridization fields. (A) An NSL-A dataset generated by manual annotation. (B) Evaluation of a series of datasets with different bounding box sizes using YOLOv5 to determine the optimal sizes. (C) NSL-B dataset with adjusted category-specific sizes. (D) Evaluation of different network architectures. (E) An augmented NSL-C dataset. (F) Determination of suitable detection models. (G) UAV images used for testing. (H) Identification of tassel states under different image blocking patterns. A1, A2, and A3 are the representative annotated samples from 3 specific categories.

A dataset of 1,000 RGB images was constructed for this study from a larger collection of over 80,000 UAV images. The selection process considered factors such as acquisition fields, maize varieties, and image quality. The dataset comprises images that captured different stages of maize growth, such as the spikelet stage, tasseling stage, and detasseling stage. These images were uniformly cropped to a resolution of 1,024 × 1,024 pixels, with a focus on the central region for detailed data annotation. The annotation process delineated the maize tassel objects into 3 specific categories: Tassel-N, Tassel-S, and Tassel-L, as shown in Fig. 1. Tassel-N refers to the central region of the plants where the tassels have been manually removed or are naturally absent. Tassel-S describes plants with tassels that have formed but not yet emerged. Conversely, Tassel-L refers to the central region of the plants where the tassels have fully emerged and reached maturity. The images were annotated by trained students using the LabelImg tool to ensure precision and consistency. Each image was methodically annotated and stored in the YOLO format for easy access and analysis. The TXT files associated with the YOLO format provide precise information, including the type of object and its relative coordinates. The information specifies the center, length, and width of each annotated bounding box.

The NSL-A annotated dataset poses a challenge due to the variable feature distributions of the maize tassels at the different growth stages. In addition, individual annotators’ subjective variances contribute to notable discrepancies in the sizes of the annotation boxes. Establishing a universally consistent labeling box for Tassel-N/S detection is difficult because Tassel-N/S often lacks clear boundaries. Furthermore, there is ongoing debate regarding the effectiveness of using a uniform box size versus an annotator-defined box size. The latter may offer more adaptability to diverse data characteristics.

To address these challenges, a rigorous evaluation of the quality of the manual annotations was conducted. Using NSL-A as a foundation, the annotation box sizes for each category were standardized on the basis of a statistical analysis of the individual category annotations. The process resulted in the creation of a dataset called NSL-B, which consisted of uniformly sized boxes. To improve the generalization capabilities and robustness of the detection models, the training data from the NSL-B dataset were augmented into the NSL-C dataset. This strategic enhancement aims to increase the reliability and effectiveness of detection models in practical applications. The data processing strategies are described as follows.

• Box size tuning: The preliminary statistical analysis of the annotated bounding boxes indicated significant variability in the dimensions across the different tassel categories. To construct a series of new datasets, the box sizes for the Tassel-N and Tassel-S categories can be incrementally expanded. Subsequently, a canonical YOLOv5 model is used to train and evaluate these datasets, thereby determining the optimized annotation size based on their detection performance. By systematically adjusting the size of the boxes and measuring the resulting precision, we can determine the annotation sizes that produce the highest accuracy for each category. These optimized bounding boxes for the tassels will ultimately be used to establish NSL-B.
• Data augmentation: It is crucial to enhance sample diversity through data augmentation to improve the model’s generalizability and robustness. The augmented NSL-C is constructed using 5 common data augmentation operations: rotation, random cropping (which alters object sizes), brightness adjustment (to mimic different lighting environments), shadow addition, and chromatic adjustment.

### Tassel detection scheme

The methodology for assessing the quality of manual detasseling in hybridization fields is shown in Fig. 1. This approach involves using UAVs to capture high-throughput images of maize canopies. These images are then used to assess the effectiveness of manual detasseling. To enable an end-to-end inference process from the UAV images to multiple semantic objects within the maize hybridization fields, a range of advanced deep learning object detection models are investigated and applied. In real-world applications, the deep learning models must be selected on the basis of specific needs to improve the model’s adaptability to complex and dynamic environments. Therefore, various deep learning models have been evaluated and optimized to achieve automatic and accurate detection and identification of maize tassels or maize plants.

The technical process comprises 3 primary stages: data acquisition, dataset generation, and model identification. In the data acquisition phase, a significant number of UAV images are collected from various maize hybridization fields, representing a range of natural lighting conditions and maize varieties. In the dataset generation phase, the NSL-A/B/C datasets (as shown in Fig. 1A, C, and E) are systematically compiled to evaluate and enhance the robustness of the tassel detection models. The model identification process involves integrating various detection models at different stages to evaluate the box size (as shown in Fig. 1B), assess the network structure (as shown in Fig. 1D), and validate the detection models (as shown in Fig. 1F). This process results in the creation of a model that accurately identifies tassels. The model is then applied to the test UAV images (NSL-T in Fig. 1G). In Fig. 1H, large UAV images are divided into blocks (patches) and batch-processed using the designed model for inference. The detection results are combined on the basis of their respective categories to perform a comprehensive semantic analysis of the UAV images to identify the validation, quantities, and locations of the tassels that are present.

### Model evaluation

Recent advancements in object detection have resulted in the establishment of standardized network architectures, which have made it easier to conduct ablation studies and benchmarks on common datasets with varying network structures and hyperparameters. Typically, modern object detection networks comprise 4 integrated modules: data processing, feature extraction, feature fusion, and prediction. The interaction of these modules enables the detectors to transform inputs into outputs through feature learning, fusion, and inference. Innovations in each module target specific challenges to improve performance. Examples include transfer learning for domain adaptation, attention mechanisms for feature refinement, and customized loss functions for improved training regulation. These advances increase the adaptability of the model to specific datasets and applications. The modular architecture of these systems provides a versatile framework for building effective object recognition models.

This study focused on tassel detection with 3 objectives: (a) to determine the optimal data size using classical models, (b) to conduct a comprehensive review of the current detection models to identify the most effective network architecture, and (c) to refine the selected models to evaluate their potential for improvement in deployment testing.

To achieve the stated objectives, we first use the YOLOv5 network on NSL-A to evaluate the impact of various size adjustment strategies. We generated the NSL-B dataset using the annotation approach, which yielded the highest accuracy in both the training and testing phases. Subsequently, we assessed several advanced CNN-based detection models, including the Faster R-CNN [17], CenterNet [19], RTMDet (real-time multitask detection) [20], and YOLOv5 to YOLOv8 [21] models. Each of these networks was applied individually to the NSL-B dataset. The model with the highest accuracy and detection efficiency [frames per second (FPS)] will undergo further evaluation to analyze its performance in different versions.

### Model training and indicators

The accuracy evaluation of the models in this study relies on several key indicators: precision (*P*), recall (*R*), mAP, FPS, number of parameters (Params), and floating point operations (FLOPs), each of which are defined by the following formulas (Eqs. 1 to 6):

$$\text{Precision}=\frac{\text{TP}}{\text{TP}+\text{FP}}$$ (1)

$$\text{Recall}=\frac{\text{TP}}{\text{TP}+\text{FN}}$$ (2)

$$\text{Accuracy}=\frac{\text{TP}+\mathrm{TN}}{\text{TP}+\text{FN}+\text{FP}+\text{TN}}$$ (3)

$$\text{IOU}=\left|\frac{A\cap B}{A\cup B}\right|$$ (4)

$$\text{AP}=\int_{0}^{1}P\left(R\right)dR$$ (5)

$$\mathrm{mAP}=\frac{1}{n}\sum_{1}^{n}\mathrm{AP}$$ (6)

where TP and FP represent the number of true positives and false positives, respectively. FN represents the number of false negatives, and TN represents the number of true negatives. *A* and *B* represent the overlap area and the union area, respectively. In Equation 6, *n* corresponds to the 3 categories of detected objects. The mAP value when the IoU (Intersection over Union) threshold is set to 0.5 is referenced to mAP@0.5.

The primary performance metrics for evaluating the deep learning models are FLOPs, Params, and FPS. The FPS reflects the number of frames (images) that an object network can process (detect) per second. Params refers to the total number of trainable parameters in a model, providing a measure of the model’s size or computational complexity. FLOPs indicate the computational demand or time complexity of an algorithm.

### Quality assessment for emasculation

This study acquired a significant number of high-resolution UAV images of maize hybridization fields. Each image has a size of 8,192 × 5,460 pixels and covers an area of approximately 280 m^2^. The acquisition objective is to extract as much information as possible, specifically the detection and location of individual tassels, while addressing the image distortions observed in the peripheral regions of the UAV images. These distortions are primarily caused by radial distortions resulting from the small-aperture imaging mechanism, compounded by the varying heights and distances of the crops from the imaging center.

To optimize image analysis performance while efficiently acquiring data, we captured UAV images with 70% heading overlap and 80% lateral overlap. Our analysis revealed that the central region of the UAV image had the least distortion and overlap with adjacent images. This study uses 3 different decomposition patterns to assess the effectiveness of reducing the data redundancy in the adjacent image sequences.

• Pattern 1: By extracting 4 image blocks with 200 pixels overlaid around the UAV image center, a square of 1,848 pixels per side (4.62 m) is created, covering an area of 21.3444 m^2^.
• Pattern 2: This involves first extracting one image block centered on the UAV image center, followed by 9 successive extractions. The resulting square formed by these 9 blocks has a side length of 2,672 pixels (6.68 m) and covers an area of 44.6224 m^2^.
• Pattern 3: This is an extraction of complete, nonoverlapping image blocks from the entire UAV image, resulting in 40 blocks arranged in an 8 × 5 grid.

The sliding window approach is a commonly used method in image decomposition. The process involves using smaller regional images for inference and then combining them with global semantic information. The important factors to consider include the size of the sliding window, the overlap rate of image blocks, and the fusion of semantic information at block boundaries. The window size selected must maintain the same pixel size as the input images of the trained model for inference consistency. The overlap rate and edge region fusion should be carefully calibrated on the basis of the statistical sizes of the detected objects. This ensures that each object is independently and completely represented in the image blocks, thus maintaining the accuracy of the object semantic fusion.

### Detection performance indicators

This study aims to assess the performance of tassel detection models based on various factors, including the number of test images, comprehensive performance metrics of the models, quantity differences between each category of detection objects, and the use of metrics such as ED (Euclidean distance), ACC (accuracy), MDR (missed detection rate), RMSE (root mean square error), MAE (mean absolute error), and MPAE (mean percentage absolute error). The model detection performance metrics are presented as Eqs. 7 to 12:

$$\text{ED}=\frac{\sum_{j=1}^{n\text{Samples}}\sqrt{\sum_{i=0}^{n}{\left({\text{Predict}}_{i}-{\text{GT}}_{i}\right)}^{2}}}{n\text{Samples}}$$ (7)

$$\text{ACC}=\frac{\sum_{j=1}^{n\text{Samples}}\frac{\sum_{i=0}^{n}{\text{Predict}}_{i}}{\sum_{i=0}^{n}{\text{GT}}_{i}}}{n\text{Samples}}$$ (8)

$$\text{RMSE}=\sqrt{\frac{\sum_{j=1}^{n\text{Samples}}{\left(\sum_{i=0}^{n}{\mathrm{GT}}_{i}-\sum_{i=0}^{n}{\text{Predict}}_{i}\right)}^{2}}{n\text{Samples}}}$$ (9)

$$\mathrm{MAE}=\frac{\sum_{j=1}^{n\text{Samples}}\text{abs}\left(\sum_{i=0}^{n}{\mathrm{GT}}_{i}-\sum_{i=0}^{n}{\text{Predict}}_{i}\right)}{n\text{Samples}}$$ (10)

$$\text{MPAE}=\frac{\sum_{j=1}^{n\text{Samples}}\text{abs}\left(\frac{\sum_{i=0}^{n}{\mathrm{GT}}_{i}-\sum_{i=0}^{n}{\text{Predict}}_{i}}{\sum_{i=0}^{n}{\mathrm{GT}}_{i}}\right)}{n\text{Samples}}$$ (11)

$$\text{MDR}=\frac{\sum_{j=1}^{n\text{Samples}}\frac{\sum_{i=0}^{n}{\text{Predict}}_{i}-\sum_{i=0}^{n}{\mathrm{GT}}_{i}}{\sum_{i=0}^{n}{\mathrm{GT}}_{i}}}{n\text{Samples}}$$ (12)

where *n* is the number of detection object categories for the model, and *n*Samples is the number of images used for testing. Predict*_i_* and GT*_i_* are the detected number and manually measured number of that category in the given image, respectively. Among these metrics, only ED evaluates the detection effect of each category individually, while the other metrics measure the overall model performance for all categories. These metrics have a common descriptive characteristic, the smaller the absolute value is, the better the model performance.

### Experimental conditions

The experimental setup was conducted on a Windows 10 platform, which was powered by an Intel Core i7-10700 CPU running at 2.90 GHz and equipped with 32 GB of random-access memory and an Nvidia GeForce RTX 1660s GPU (graphics processing unit) with 6 GB of memory.

PyTorch version 1.8.2 was used for the deep learning framework, coupled with Compute Unified Device Architecture 10.2, and optimized with cuDNN version 8.0.5. The mmyolo repository (available at https://github.com/open-mmlab/mmyolo) was used to implement various YOLO and RTMDet models. For other models, such as the CenterNet and Faster R-CNN models, the mmdetection toolkit (https://github.com/open-mmlab/mmdetection) was used. The models were trained using default hyperparameters, with a batch size of 8 for single GPU processing.

## Results

### Determination of optimal annotation strategies for tassels

**Fig. 2.**
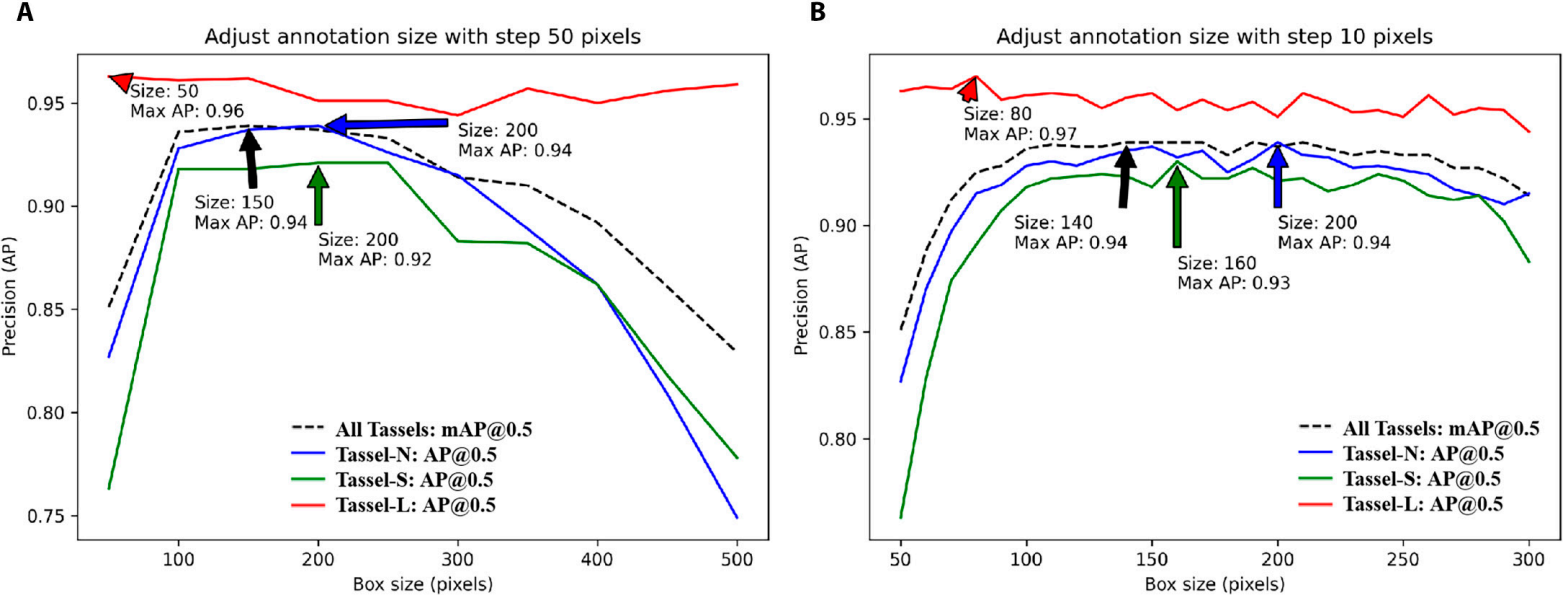
Determining the box size of the tassel objects by evaluating the accuracy of the detection models. (A) Increments of 50 pixels and (B) increments of 10 pixels.

Properly setting the annotation box size for each maize plant is crucial to force the deep learning models to focus on, as well as extract, the central features of the tassels. However, directly determining the optimal box size is difficult. Therefore, setting a uniform annotation box size centered on the plant could help standardize the semantic description of the tassels. This study aims to adjust the annotation box sizes for 2 categories of tassel objects, Tassel-N and Tassel-S, by incrementally modifying the sizes through enumeration schemes. The dataset was then divided into training, validation, and test sets at an 8:1:1 ratio. The YOLOv5 model was trained using the training and validation sets, and the model performance for each object category was evaluated using the test set. The relationship between detection performance and box size was analyzed to identify the optimal annotation dimensions of the tassel objects, as demonstrated in Fig. 2.

Box sizes were adjusted in increments of 50 pixels within the range of 50 to 500 pixels (Fig. 2A). The highest precision, 0.94 for Tassel-N and 0.92 for Tassel-S, was achieved when the annotation box lengths were 150 and 200 pixels, respectively. The model’s overall detection precision for all categories initially increased and then decreased, indicating a significant effect of the annotation box size on model precision. This theoretically allows for the determination of an optimal annotation dimension. Therefore, we refined the annotation box increment to 10 pixels within the range of 50 to 300 pixels to determine the dimensions that yielded the highest detection precision for both Tassel-N and Tassel-S, as shown in Fig. 2B. The detection accuracy for the 3 categories remained stable when the box dimensions were between 100 × 100 pixels and 220 × 220 pixels. The model achieved peak detection accuracies of 0.94 for Tassel-N and 0.93 for Tassel-S when the annotation box sizes were 200 and 160 pixels, respectively.

We also observed slightly fluctuating detection accuracies for the unadapted Tassel-L objects in the test set. The Tassel-L category achieved the highest detection precision (0.97) when the annotation box sizes for Tassel-N and Tassel-S were 80 pixels. In multiobject detection models, the precision of detecting the different categories may be interdependent. This is due to the loss calculation of the detection model, which balances the relationships between different categories, resulting in nonindependent detection results for each category. However, this effect was not found to be significant in this experiment. On the basis of Fig. 2B, the detection model achieved its highest detection precision when the numbers of annotated boxes of Tassel-N, Tassel-S, and Tassel-L were set to 200, 160, and 80 pixels, respectively.

In summary, our comprehensive analysis of how the size of the annotation boxes affects the object detection accuracy in maize hybridization fields yields several key findings:

• Annotations determine model performance: We found only small variations in detection accuracy across all 3 categories when the annotation box sizes were between 100 and 240 pixels. This suggests that within this specified range, variations in the annotation box size have minimal effects on the detection accuracy.
• Potential interactions between category annotations: Changing the dimensions of the annotation boxes for certain objects unexpectedly affected the detection accuracy of other objects with unadjusted box sizes. This observation highlights the profound impact that changes in annotation box sizes have on the accuracy of object recognition models.
• The annotation size can be optimized for categories with unclear boundaries: If the box size is too large, it could lead to an increased overlap between adjacent object annotations or even a single box covering multiple objects, misleading the model, and generating numerous duplicate recognition results. Conversely, if the box size is too small, CNNs may struggle to learn enough features, resulting in reduced detection accuracy. Furthermore, in complex maize fields, leaf occlusion is a significant factor in the loss of tassel information. If the box is too small, many slightly occluded objects may be missed. Therefore, these factors need to be considered when setting the box size to ensure that the model accurately extracts tassel features and improves detection accuracy.
• The size of the annotation is related to the actual situation. In real-world scenarios, the distance between maize plants typically remains within a consistent range, such as 25 cm for plant spacing and 60 cm for row spacing. Standard maize planting provides guidelines for determining the appropriate annotation box dimensions. The above experiments suggest that the annotation box sizes (ranging from 80 to 150 pixels for Tassel-N/S) are well aligned with these agricultural norms.

To accurately assess the performance of the different detection models, we use images with annotation box sizes of 140 pixels for training purposes. The proposed approach aims at minimizing errors due to annotation box size adjustments, thereby providing a more robust basis for subsequent model optimizations. Furthermore, we anticipate that this annotation strategy will encourage further research focused on the impact of the annotation box dimensions on object detection accuracy for some specific scenarios, thereby contributing to the advancement of object detection technologies.

### Statistical analysis of the annotation datasets

On the basis of the evaluation using the tassel annotation sizes described above, we further generated 2 annotated datasets for the evaluation of the tassel detection models. The manually annotated dataset is called NSL-A, the dataset with adjusted annotation box sizes is called NSL-B, and the extended dataset based on NSL-B is called NSL-C.

**Fig. 3.**
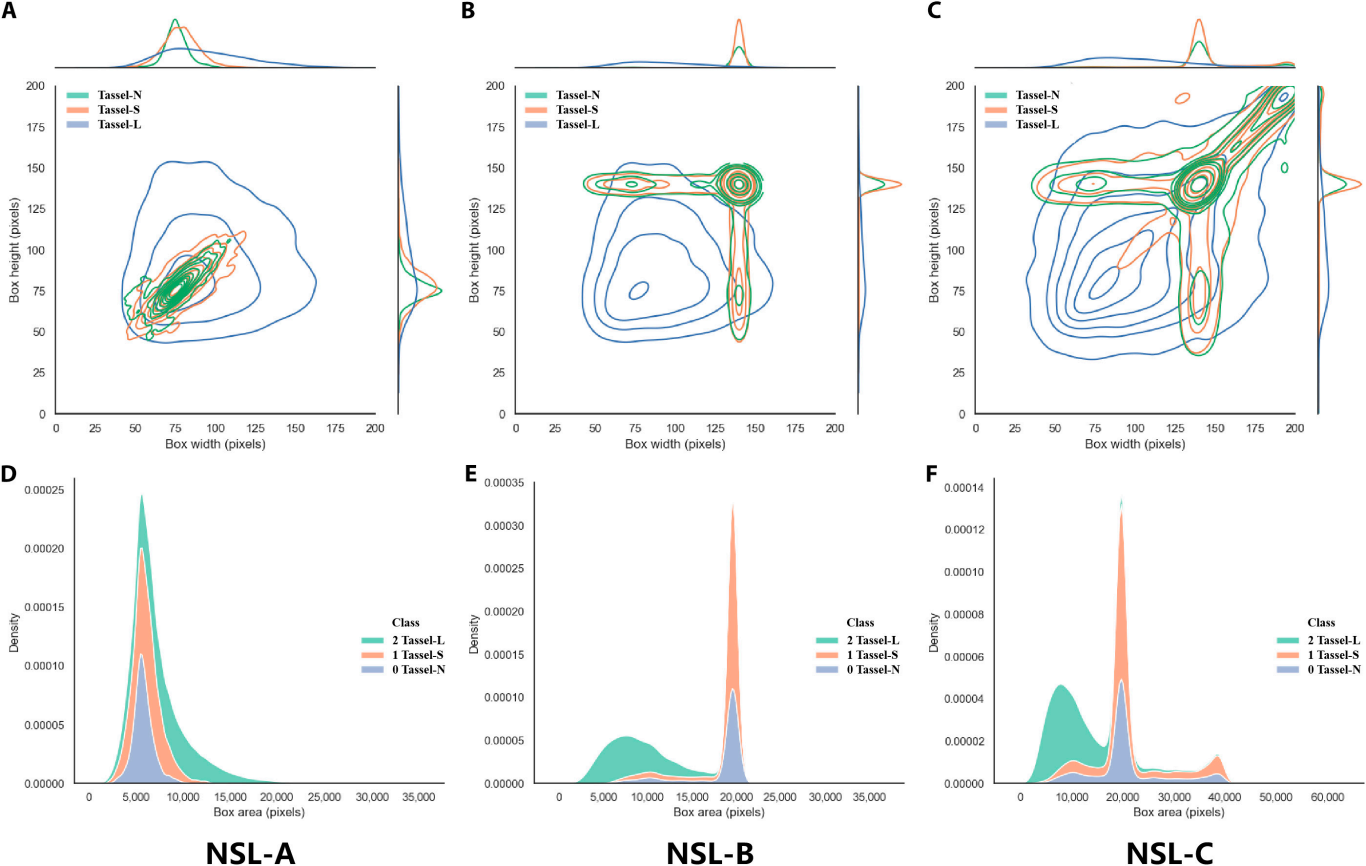
Distribution of bounding box sizes for the tassel categories. (A to C) The width and height distribution annotation boxes for the NSL-A/B/C datasets. (D to F) The distribution densities of the tassel N/L/S categories for the NSL-A/B/C datasets.

The distribution of label sizes in the NSL-A/B/C datasets is shown in Fig. 3. The NSL-A dataset, which was collaboratively annotated by multiple individuals, shows a normal distribution in the tassel annotation area, with an average box area of 5,784 pixels and an average side length of approximately 76 pixels, as shown in Fig. 3A and D. In the NSL-A dataset, we documented the number of tassels in each category as follows: Tassel-N (40.93%), Tassel-S (35.24%), and Tassel-L (23.83%), for a total of 29,114 annotation boxes.

After adjusting the annotation box sizes of Tassel-N and Tassel-S to 140 pixels, the statistical distribution of the resulting NSL-B dataset, as shown in Fig. 3B and E, remained consistent in terms of the number and proportion of each category compared to those of NSL-A. However, the size and distribution of the annotation boxes changed significantly.

The NSL-B training subset of 800 images was augmented, resulting in the augmented NSL-C training dataset, which ultimately contained 4,200 training images. This dataset contained 137,938 annotation boxes (Fig. 3C and F), with the object proportions being Tassel-N (41.11%), Tassel-S (33.88%), and Tassel-L (25.01%).

Table 1 shows the statistical information of the annotation boxes in the 3 datasets. The Tassel-L category, with clear boundaries and unadjusted sizes, showed relatively consistent box areas (except for the size changes due to a rotation argumentation operation for NSL-C), with a length–width ratio averaging between 0.75 and 0.76 and a variance of 0.16 to 0.18 across all 3 datasets. The other 2 categories, i.e., Tassel-N and Tassel-S, underwent significant size adjustments, with length–width ratios above 0.92. Furthermore, in the NSL-C dataset, the mean annotation area for each category was remarkably consistent, at 11,039, 10,833, and 10,129 pixels, respectively. However, the variance in the annotation box sizes for each category in the NSL-C dataset was significantly greater than that in the first 2 datasets.

**Table 1.** Statistical analysis of 3 categories of annotation boxes for 3 datasets

Datasets	Box features	Tassel-N		Tassel-S		Tassel-L		All tassels	
		Mean	SD	Mean	SD	Mean	SD	Mean	SD
**NSL-A**	Box area (pixels)	5,784	1,186	6,256	1,500	8,647	3,735	7,122	2,964
	Length–width ratio	0.95	0.06	0.94	0.07	0.75	0.16	0.87	0.15
**NSL-B**	Box area (pixels)	9,436	1,338	9,611	1,125	8,647	3,735	9,175	2,604
	Length–width ratio	0.94	0.13	0.96	0.11	0.75	0.16	0.87	0.17
**NSL-C**	Box area (pixels)	11,039	4,364	10,833	3,454	10,129	5,823	10,595	4,785
	Length–width ratio	0.92	0.17	0.95	0.14	0.76	0.18	0.86	0.19

These datasets can also be used as a reference when creating annotation datasets that are more aligned with real-world applications and can also be used to identify appropriate feature extraction network architectures and recognition models.

### Evaluation of the detection network architecture

A wide range of CNN models are widely used in image recognition, including the YOLO series, Faster R-CNN, RTMDet, and CenterNet. These models have shown consistent improvements in accurate image recognition and adaptation to different data types. However, there is no single CNN model that is perfectly suited to all recognition tasks; the choice depends on the specific requirements of each task.

**Fig. 4.**
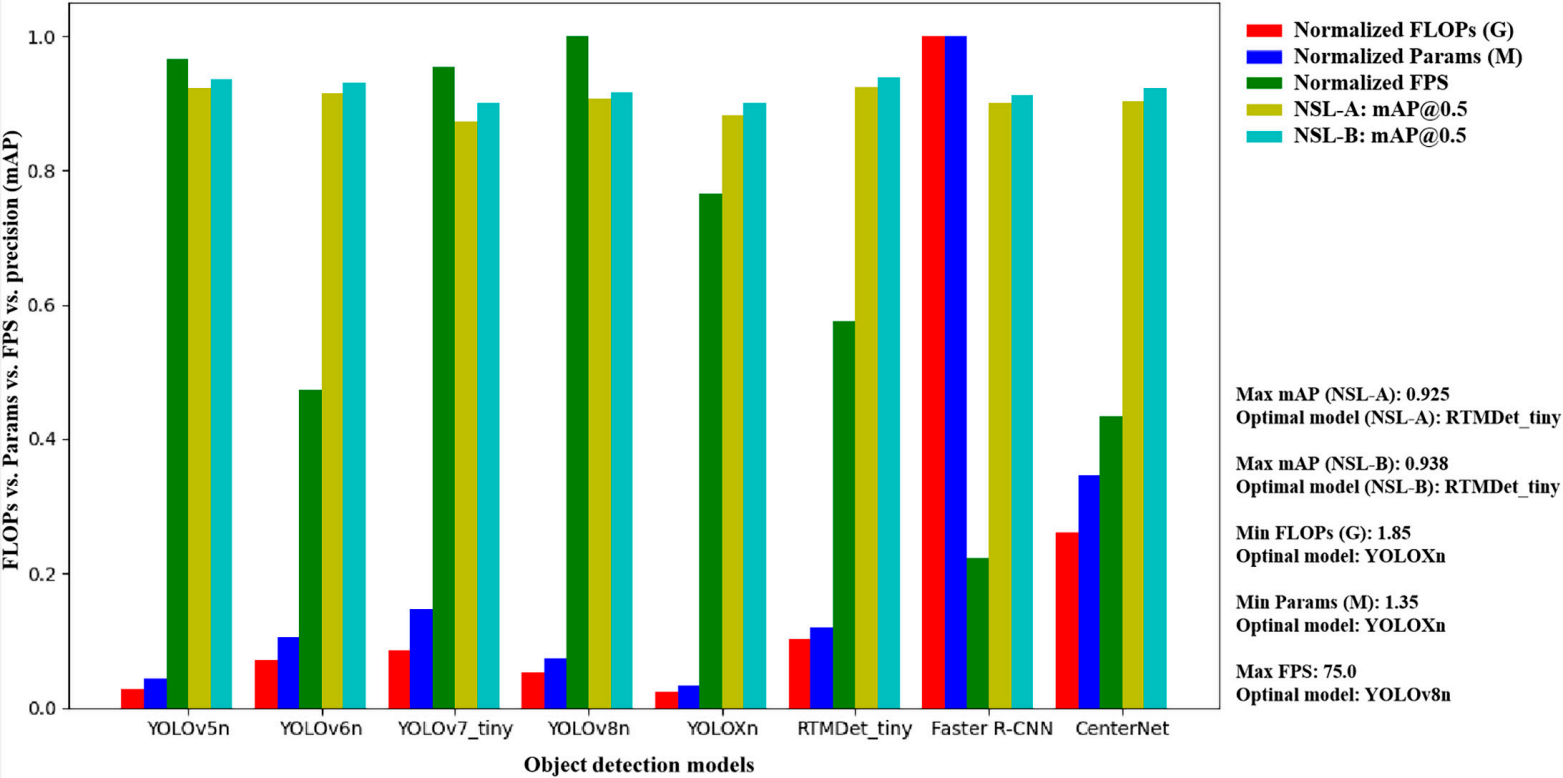
A comprehensive evaluation of object detection models is performed on the basis of the metrics of FLOPs, Params, efficiency (FPS), and accuracy (mAP@0.5).

This study evaluates the performance of these models on 2 different datasets, the original NSL-A dataset and the adapted NSL-B dataset. Smaller but more effective versions of these models were chosen to make the training process faster and more efficient. The evaluation focuses on the mAP@0.5. These factors were normalized for a thorough comparison, as shown in Fig. 4. The models trained on the NSL-B dataset perform better than those trained on the NSL-A dataset, highlighting the value of having well-prepared and consistent data for model training. In particular, the RTMDet model shows a high detection accuracy of 93.8% on NSL-B and 92.5% on NSL-A, indicating a 1.3% improvement in accuracy due to better annotation strategies. In addition, although the YOLOXn and YOLOv8n models have advantages in terms of the FLOPs, Params, and FPS metrics and exceed a 90% accuracy, RTMDet achieves the highest accuracy on the NSL-A/B datasets.

**Fig. 5.**
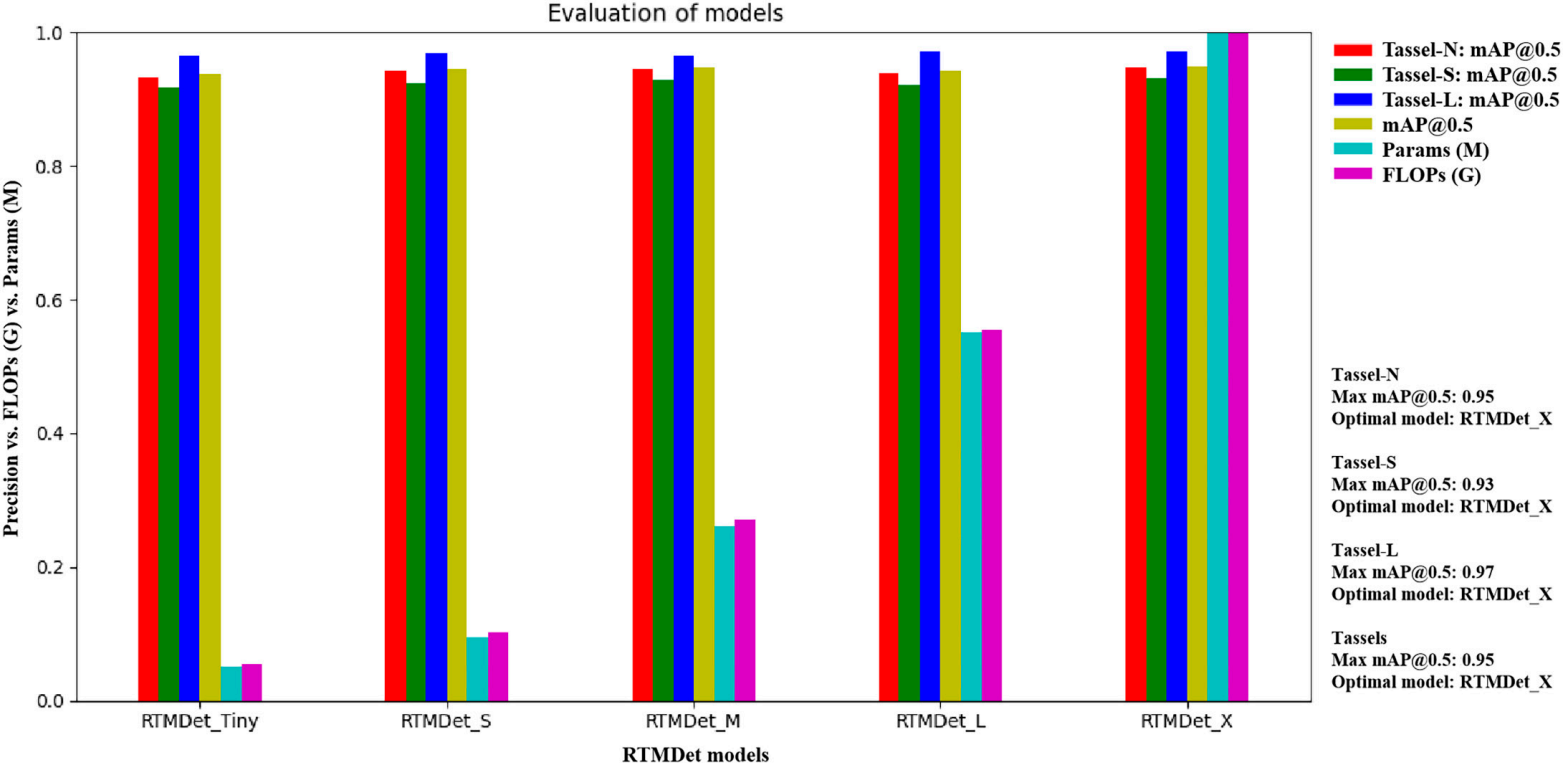
Evaluation of RTMDet models with different parameter scales based on the NSL-C dataset. Note that the evaluation indicators consist of FLOPs, Params, efficiency (FPS), and precision (mAP@0.5) of the 3 tassel categories.

In real-world applications, the choice between these models involves balancing their computational and memory requirements to suit the specific situation. For example, the Faster R-CNN model, with its high computational and memory requirements, may not be ideal for devices with limited computational and memory resources. On the other hand, YOLOXn, which requires less power and memory, is not as accurate and may not be suitable for detection tasks with high accuracy requirements. From a computational efficiency perspective, YOLOv8n achieved the highest 75 FPS but had slightly lower detection accuracies of 91.7% (NSL-B) and 90.6% (NSL-A). Therefore, in cases such as the detection of maize tassel states, as investigated in this study, a model with high accuracy, especially for detecting Tassel-S, is recommended. The RTMDet model, known for its accurate and real-time detection, has emerged as the preferred choice for such applications.

### Identification of the optimal RTMDet model

On the basis of an analysis of the computational resource requirements, detection accuracy, and inference speed, an appropriate object detection model was selected for practical application. The first step was to identify the computational resources required to support model deployment. Models with larger parameter sizes always require more computational resources, such as GPU memory and processing power. Next, the issue of object recognition accuracy was assessed. Typically, the model with the highest mAP@0.5 was selected; however, it was found that in multiobject recognition scenarios, there can be significant differences in recognition accuracy between different object categories. Finally, the inference speed of the model was considered. Particularly in situations requiring real-time responses, the inference speed of the models becomes a critical factor within the current computational resource restraints and acceptable levels of accuracy.

Therefore, an evaluation of different parameter-scaled versions of the RTMDet network model was conducted to select one that meets the current practical application and computational resource constraints. The RTMDet includes pretrained models of the Tiny, S, M, L, and X versions, with progressively increasing parameters and FLOPs, as shown in Fig. 5. Each of these models was trained on the NSL-C dataset, and their performances are shown in Table 2. The average detection accuracy of the 5 versions of the RTMDet model differed by a maximum of only 1.1%, with the largest differences in detection accuracy for the Tassel-N/S/L categories being 1.4%, 1.4% and 0.7%, respectively. The X version, with the largest parameter size, almost always achieved the best detection performance, except for the L version, where it achieved the highest average detection accuracy for the Tassel-L category at 97.1%. However, these models with different parameter sizes show an almost exponential increase in FLOPs and Params, with a corresponding incremental increase in detection performance (e.g., FPS). Considering all these factors, the S version of the RTMDet network model was selected for tassel detection in this study.

**Table 2.** Comparative analysis of the detection performances of the RTMDet models with different parameters

RTMDet models	AP:Tassel-N	AP:Tassel-S	AP:Tassel-L	mAP@0.5	FLOPs (G)	Params (M)
RTMDet_Tiny	0.933	0.917	0.964	0.938	8.030	4.870
RTMDet_S	0.943	0.923	0.968	0.945	14.750	8.860
RTMDet_M	0.946	0.930	0.964	0.947	39.080	24.670
RTMDet_L	0.940	0.921	0.971	0.944	79.960	52.260
RTMDet_X	0.947	0.931	0.970	0.949	144.380	94.780

### Model evaluation for different growth stages

We have previously evaluated and identified the RTMDet architecture for tassel detection applications. Therefore, we trained corresponding RTMDet_S models on the NSL-A, NSL-B, and NSL-C datasets, resulting in 3 versions of the models corresponding to the datasets, RTMDet-NSL-A, RTMDet-NSL-B, and RTMDet-NSL-C. These models were then used to evaluate their accuracy and performance in detecting tassels in UAV test images from the different tassel growth stages. For this study, the NSL-T dataset, which is independent of all the image datasets used for model training, was constructed. These test images (30 images in total) were taken at 3 different maize growth stages—the spikelet stage, tasseling stage, and detasseled stage—with 10 images used for each stage. The number of objects in each tassel category was counted manually. The total numbers of objects in the 3 growth stages were 16,600, 13,679, and 11,564, respectively, which are the ground-truth values for our quantitative evaluation.

**Fig. 6.**
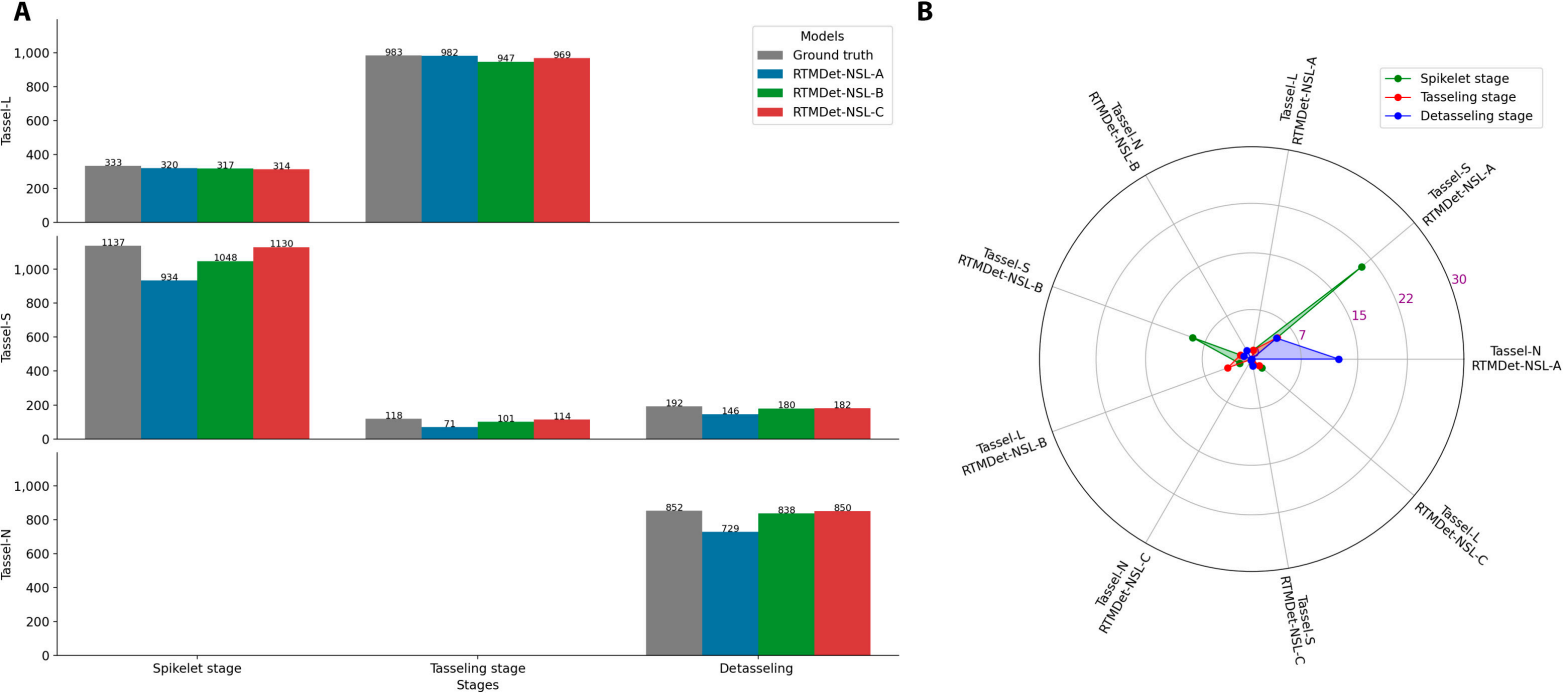
Quantitative comparison of the detection results of the 3 tassel categories in the test images of the NSL-T dataset. (A) Quantitative comparison of object detections between models for each tassel category. (B) Overall evaluation of the tassel categories for the different detection models.

From each UAV test image, 4 image blocks of 1,024 × 1,024 pixels were extracted for model inference. These image blocks were derived from the central region of each UAV test image and covered the 1,848 × 1,848-pixel central region (i.e., the adjacent blocks with a 200-pixel overlap between blocks). The number of ground-truth tassel objects manually counted across the test images from the 3 stages, as well as the prediction results using the 3 models, are shown in Fig. 6. Figure 6A shows that the number of objects in the tassel categories differed significantly among the different growth stages, with Tassel-S predominating during the spikelet stage, Tassel-L predominating between the tassel emergence to maturity stages, and Tassel-N predominating during the detasseled stage. In addition, we note that models built on the 3 datasets show differences in performance on the test images from the different growth stages, making it difficult to identify an optimal model that can best process images across all the growth stages.

For the maize growth stage with predominantly Tassel-S, RTMDet-NSL-A produces results closest to the ground truth, while for scenarios with predominantly Tassel-L and Tassel-N, RTMDet-NSL-C performs better. To comprehensively evaluate the performance of the 3 models, the differences in tassel categories between the predicted and ground-truth values are used to describe the overall performance, as shown in Fig. 6B. The experimental results show the following:

• At the spikelet stage, the detection accuracies of RTMDet-NSL-A/B/C for Tassel-S were 82.1%, 92.2%, and 99.4%, respectively, while those for Tassel-L were 96.1%, 95.2%, and 94.3%, respectively. Moreover, the overall detection accuracies of the 3 tassel categories are 85.3%, 92.9%, and 98.2%, respectively. Therefore, for the UAV images from this stage, an approach combining the 2 models could be considered, i.e., using RTMDet-NSL-C to detect the Tassel-S category and RTMDet-NSL-A to detect the Tassel-L category.
• At the tasseling stage, only the Tassel-S/L categories remain, and Tassel-L predominates over Tassel-S. Here, the detection accuracies of RTMDet-NSL-A/B/C for Tassel-S are 60.1%, 85.6%, and 96.6%, respectively, while for Tassel-L, they are 99.9%, 96.3%, and 96.6%, respectively. The overall performances are 95.6%, 95.2%, and 98.4%, respectively. This seems to be in line with the conclusions of the previous stage.
• At the detasseled stage, the Tassel-N category is present in the UAV images, while Tassel-L is absent (having been manually detasseled). The RTMDet-NSL-A/B/C detection accuracies for Tassel-N were 85.3%, 98.4%, and 99.8%, respectively, while those for Tassel-S were 76.0%, 93.8%, and 94.8%, respectively. The overall performance indices are 83.8%, 97.5%, and 98.9%, respectively. For UAV images from this scenario, RTMDet-NSL-C remains the optimal model choice.

In summary, when the UAV images contain only Tassel-S/L, the RTMDet-NSL-C model is always able to detect Tassel-S more accurately, with an improvement in detection performance on Tassel-S of 17.2% to 36.4% compared to RTMDet-NSL-A, and 7.2% to 11.0% compared to RTMDet-NSL-B. It should be noted that RTMDet-NSL-A always achieves higher detection accuracy on Tassel-L, outperforming the RTMDet-NSL-B model by 0.9% to 3.6% and the RTMDet-NSL-C model by 1.3% to 1.8%. This shows that for a tassel category with clear boundary features, models based on real boundary annotations can achieve higher accuracy. For multiobject detection models, adjusting the annotation size of one category not only can improve the detection accuracy but also introduces uncertainty that affects the detection results of other objects (manifested here as improved accuracy for Tassel-S but slightly reduced accuracy for Tassel-L). For models trained with data augmentation, Tassel-S detection reached a high accuracy of 99.4%, but the Tassel-L detection accuracy became somewhat unstable.

Furthermore, when UAV images contained only Tassel-N/S, both the size-adjusted and data-enhanced models showed gradual improvements in detection accuracy. This shows that both strategies proposed here are highly effective. For such scenarios, the RTMDet-NSL-C model remains the optimal choice, improving the Tassel-N detection accuracy by 14.2% and 1.41%, respectively, over that of the RTMDet-NSL-A/B models and improving the Tassel-S detection accuracy by 18.8% and 1.0%, respectively. Comparatively, the size adjustment strategy contributes more significantly to improved detection accuracy for both tassel categories, while the data augmentation strategy provides a slight further improvement.

### Model evaluation for different blocking patterns

To verify the detection performance of the different image blocking strategies on maize tassel states when processing the actual UAV images acquired from maize hybridization fields, we investigated how to make the most of the information in a single UAV image while maintaining tassel detection accuracy. In general, the larger the effective usable area in a single UAV image is, the more efficient the use of UAVs for acquisition and subsequent image analysis will be. This can provide valuable guidance for the development of efficient flight plans and data acquisition procedures. The high-resolution P1 camera used in this study can capture and cover a large area, but, strictly speaking, only the central area of the captured image can be considered the approximate true orthographic area. On the basis of the imaging principle of perspective projection, this image distortion effect caused by optical pinhole imaging is also known as perspective distortion. Specifically, for scenes photographed perpendicularly, the tassels further away from the central axis of the image are more likely to be photographed by the camera’s imaging surface from their sides or oblique surfaces than from the tassel canopy. It can be observed that plants and their tassels further away from the lens center appear slenderer in the image, and the degree of distortion is proportional to the distance of the object from the center of the image, with greater distortion at greater distances. This effect is particularly pronounced for wide-angle lenses and can also be clearly observed for the 35-mm lens used in this study, especially at lower UAV flight altitudes.

**Fig. 7.**
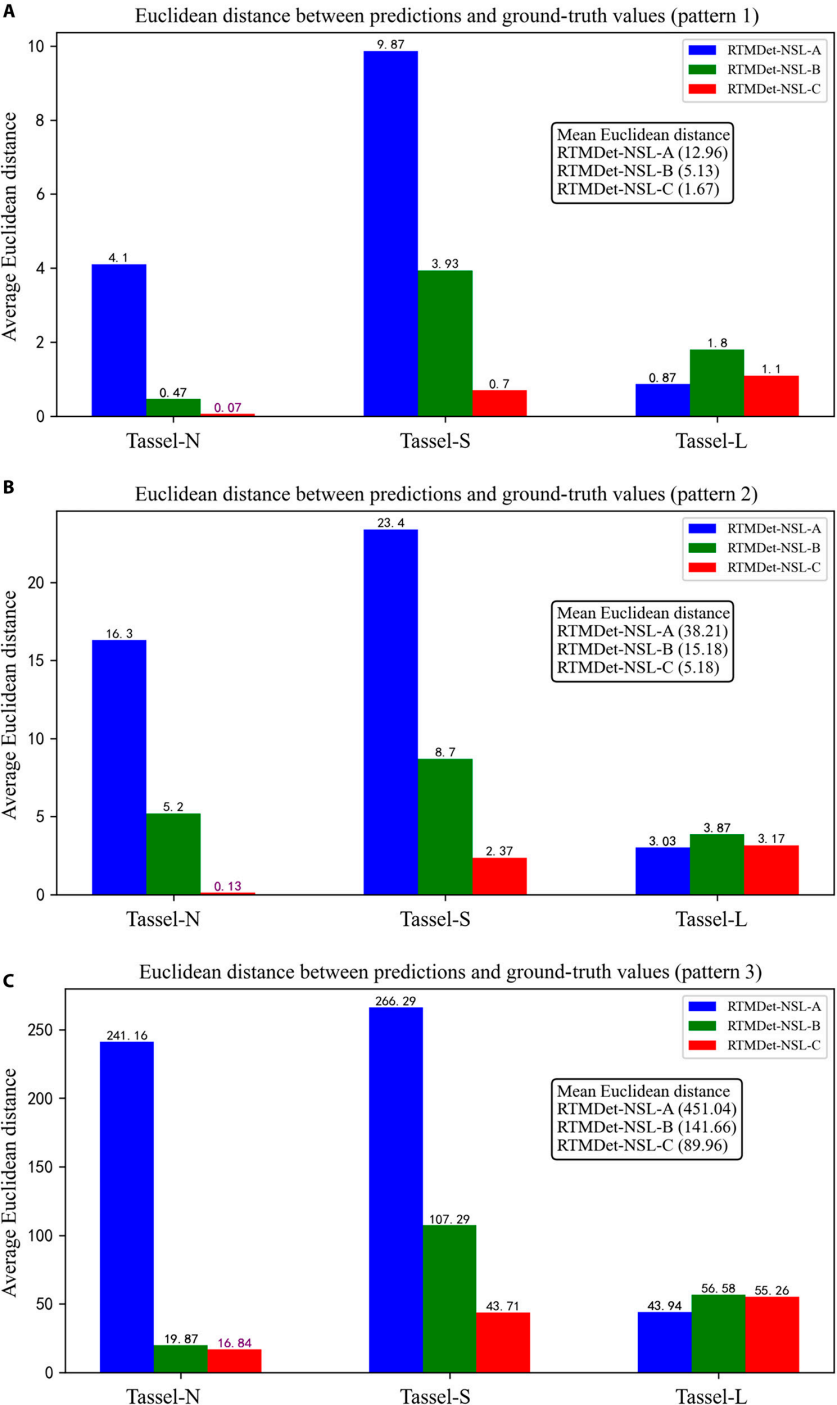
Evaluation of model performance (RTMDet-NSL-A/B/C) using EDs between predictions and ground-truth values under 3 blocking patterns (patterns 1 to 3). (A) For test images acquired at the spikelet stage. (B) Test images acquired at the tasseling stage. (C) Test images acquired at the detasseling stage.

To assess the impact of such image distortions on tassel detection, we tested and validated 3 image blocking strategies that decompose single UAV images into 1,024 × 1,024 overlapping image blocks around the image center. Blocking patterns 1 and 2 extract 4 and 9 central image blocks, respectively, while pattern 3 can extract more than 40 image blocks. In the previous section, we evaluated the detection performance of the different tassel stages under blocking pattern 1. Here, we further evaluate the model performance under 3 blocking patterns to provide a reference for quantitatively assessing the impact of blocking patterns on the final tassel detection accuracy. In addition, we uniformly apply the ED formula to evaluate the detection performance for different tassel categories, as shown in Fig. 7. Across the NSL-T dataset (regardless of the tassel growth stage), the performances of all 3 detection models deteriorate progressively from patterns 1 to 3. As shown in Fig. 7A to C, the average ED metric of the RTMDet-NSL-C model remains the lowest across all block patterns at 1.67, 5.18, and 89.96, respectively, indicating that it maintains an optimal performance for each block pattern, despite an unacceptable performance degradation for pattern 3. To allow a fair comparison despite the significantly different number of blocks for the 3 patterns (4, 9, and 40 blocks, respectively), the ED per block can be recalculated to be 0.42, 0.57, and 2.25, respectively. This indicates that increasing the number of image blocks around the center of the image does indeed have a significant effect on the recognition accuracy of these models.

In blocking pattern 1 (Fig. 7A), RTMDet-NSL-C shows significantly greater detection accuracy for the Tassel-N/S targets than do the other models. The ED reached 0.07 and 0.7 for Tassel-N, indicating quantity errors within one object. In addition, RTMDet-NSL-A shows excellent performance in detecting the Tassel-L category, with an ED of 0.87, which is also within one object. For blocking patterns 2 and 3 (Fig. 7B and C), the relative changes between the models remain similar to those for pattern 1, but the detection accuracy deteriorates significantly. This suggests that the blocking pattern needs to be determined on the basis of object detection accuracy requirements and that distal regions far from the image center are not suitable for tassel detection and analysis.

### Comprehensive performance assessment

In particular, the models used here are all trained on images extracted from the central region of the UAV images. Therefore, using these models to evaluate all the image blocks extracted from the entire image is not entirely fair. In any case, accurate detection of tassel semantics from massive UAV images currently requires the use of densely captured UAV image sequences to compensate for the detection accuracy, which will be an issue to be addressed in future studies.

Table 3 lists comprehensive performance evaluation metrics for the models under the 3 blocking modes. A higher ACC indicates higher model accuracy in tassel identification and counting. The MPAE is another metric that reflects differences between predicted and actual values, with the lower values indicating a higher model prediction accuracy. The ACC and MPAE metrics show that RTMDet-NSL-C has significant advantages over the other 2 models, reaching 98% for blocking patterns 1 and 2 and 93% for pattern 3.

**Table 3.** The model evaluation indicators for blocking patterns 1 to 3 for NSL-T

Blocking pattern	RTMDet models	ED	ACC	MDR	RMSE	MAE	MPAE
Pattern 1 (4 blocks)	RTMDet-NSL-A	3.14	0.88	−0.12	17.22	14.5	0.12
	RTMDet-NSL-B	1.42	0.95	−0.05	7.80	6.13	0.05
	RTMDet-NSL-C	0.52	0.98	−0.02	2.83	1.87	0.02
Pattern 2 (9 blocks)	RTMDet-NSL-A	8.52	0.84	−0.16	46.67	42.27	0.16
	RTMDet-NSL-B	3.54	0.93	−0.07	19.38	17.77	0.07
	RTMDet-NSL-C	1.43	0.98	−0.02	7.84	5.56	0.02
Pattern 3 (40 blocks)	RTMDet-NSL-A	180.27	0.7	−0.3	1003.67	551.39	0.3
	RTMDet-NSL-B	41.18	0.89	−0.11	229.26	178.71	0.11
	RTMDet-NSL-C	26.08	0.93	−0.07	145.22	114.06	0.07

The RMSE and MAE metrics describe the deviation of the model predictions from the ground truth from a quantitative perspective and show similar trends to those of the ED, with lower values being preferred. Note that the number of image blocks for each pattern is significantly different when evaluating these 3 metrics. The MDR values are negative floating point values, indicating that there are fewer predicted objects than actual objects, so these models can be further improved by adding more training data to better capture the features of the tassels to be detected. It is worth noting that lowering the object screening threshold of the models can also improve the MDR.

### Tassel assessments for UAV imagery

Note that in the figure, red, green, and blue cross-markers denote Tassel-S/L/N, yellow denotes the central reference region of the UAV image, purple denotes different image partitioning patterns, cyan denotes representative image blocks, and the white circles and diamond markers denote the corrected objects that merged redundant tassels at a predefined distance (here, 50 pixels were used).

**Fig. 8.**
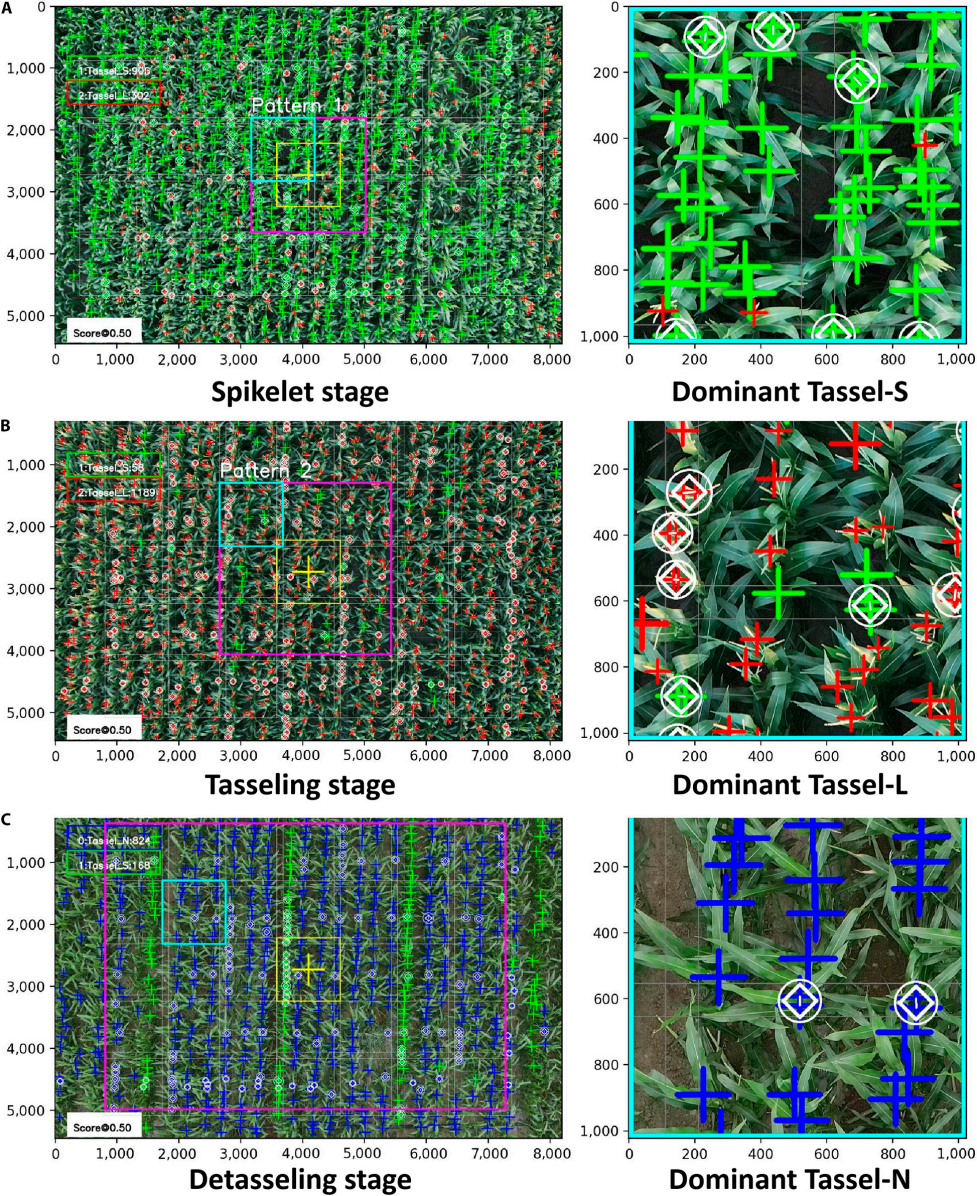
Detection and evaluation of the tassel states at 3 growth stages in maize hybridization fields. (A) Spikelet stage (Tassel-S category is numerically dominant, although there is also sporadic emergence of the Tassel-L category). (B) Tasseling stage (Tassel-L starts to emerge widely, occupying most of the space). (C) At the bypassing stage, large numbers of Tassel-N plants are detected in the manually labeled maternal maize plants, while the paternal plants remain at the Tassel-S and Tassel-L stages.

To maintain model prediction accuracy, large UAV images must be cropped into appropriately sized image blocks before being fed into the detection models. These image blocks, which are derived from the source UAV image, can be batch-processed through the detection model. Subsequent processing includes repositioning and semantic unification of the detection results. The efficiency of a UAV image analysis is directly determined by the image blocking strategy. In this study, the RTMDet-NSL-C model is used to quantitatively evaluate the NSL-T dataset, as shown in Fig. 8. The analysis results of the tassel states at different growth stages of maize (spikelet, tasseling, and detasseling) are shown in detail in Fig. 8A and B, which also shows the analysis results of the 3 blocking patterns. The tassel numbers of the 3 tassel categories varied considerably over the different stages of maize tassel development. This makes it possible to predict the growth stage of tassels based on their number and states.

Figure 8A shows the spikelet stage, which consists mainly of the Tassel-S category with sparse tassel emergence. This stage is crucial for manual detasseling in maize seed production because it requires the removal of most of the female tassels before pollen release. At this stage, the Tassel-S objects are the most numerous, although occasional Tassel-L objects appear. When analyzing the image block under the first blocking pattern, an orthographic perspective of the maize canopy appears to be densely populated with the Tassel-S objects, necessitating a merge count due to their close proximity.

Figure 8B shows that Tassel-L objects predominate during the tasseling stage, while a few Tassel-S objects have yet to emerge because of uneven plant growth. Accurate discrimination of the Tassel-S objects becomes essential. The image block from the second blocking pattern, which is situated slightly away from the image center, shows different slopes in both the maize plants and the tassels. This shows the changes in the position of the blocks relative to the center of the image, which adversely affects the accuracy of the detection model. Figure 8C shows a detailed scenario in maize hybridization fields, where the female tassels are largely removed and the tassels of the male plants are in the Tassel-S/L stages. The detection of missed tassels on female plants is both critical and challenging.

In addition, the existing detection models tend to miss objects in image blocks situated away from the image center because the models are primarily trained on the cropped central regions of UAV images. The overlapping areas of the image blocks often result in redundant tassel objects. To address this issue, we merge objects based on their category and location. In this case, we use a 50-pixel threshold to merge objects in close proximity; the emphasis is marked by white circles and diamonds.

The experiment supports the effectiveness of the proposed method in identifying and analyzing the different tassel states in UAV images. It achieves 98% accuracy in identifying Tassel-S in the first blocking pattern. These tassel states provide evidence for determining the tasseling stage of the maize. In maize hybridization fields, the staggered planting of male and female plants at different times regulates their flowering periods. Therefore, the tassel semantics parsed from the UAV images can be assigned valuable meanings related to each tassel stage. As a result, the category and number of tassels in the UAV images can be used to delineate the growth stages of the male and female maize plants. For example, if a UAV image shows a predominance of Tassel-S objects and few Tassel-L objects, manual detasseling of the female plants is needed. A predominance of Tassel-N objects indicates that at least one round of preliminary manual detasseling has been carried out. It is important to detect and identify any missing Tassel-S/L objects in the female plant area to formulate subsequent manual detasseling plans. Analysis of the different tassel states in UAV images can assist field managers in recognizing the growth dynamics of the maize tassels. This can help formulate targeted and efficient management practices.

## Discussion

This study presents an innovative methodology for assessing maize tassel states in maize hybridization fields using drone imagery, incorporating several key improvements. A key aspect of our approach is the investigation of the influence of the annotation size on the tassel detection models. This investigation provides a theoretical basis for similar future annotations and evaluates the efficiency of data augmentation techniques, the maximization of data mining potential, and the strengthening of model resilience and adaptability.

In terms of data annotation, we advocate an approach that ensures that annotations accurately reflect the true contours of dynamically evolving entities such as tassels. We recommend uniform annotation sizes for these variable objects, with the ideal dimensions derived from the distribution and proximity of the objects within the scene. Our methodology clearly categorizes 3 types of annotated targets: Tassel-L, Tassel-N, and Tassel-S. While the Tassel-L objects are easily identifiable and accurately annotated, the Tassel-N and Tassel-S objects initially lack definitive annotation guidelines. After evaluating a range of sizes, we determined an optimal annotation size spectrum. The initial dataset, NSL-A, was constructed to minimize overlap between bounding boxes centered on maize plants, which was followed by adjustments to the Tassel-N and Tassel-S sizes in the NSL-B dataset using square bounding boxes. The effectiveness of the different annotation sizes was then determined by training different detection models using the YOLOv5 model for validation, thus demonstrating the effectiveness of our targeted annotation strategy.

In preparing the datasets, we considered contributions from multiple annotators, optimized the dataset size, and implemented data augmentation. Training similar models with each dataset variant underlined the indispensable role of data quality in achieving precise detection goals. These strategies provide guidance for further refinement of model accuracy.

For model selection and evaluation, despite progress in deep-learning-based object detection models, further significant breakthroughs remain possible. The use of mature, industry-proven frameworks may be sufficient for current applications. However, the effectiveness of these frameworks is highly variable, largely because of nonstandardized and incomplete data preparation. Prioritizing the acquisition of real-world data and the extraction of object information for model training are paramount to the development of valuable models.

When evaluating detection performance, we considered variations in the application scenarios and the interactions between the detection accuracies of the different models. The performance of models trained on different datasets varied across scenarios. Although RTMDet-NSL-C typically showed a superior performance, it was slightly outperformed by RTMDet-NSL-A in the detection of Tassel-S. The quest to train a universally exemplary model requires extensive ablation studies at increased cost and effort. A more pragmatic approach may be to exploit the collective strengths of the different models.

In terms of UAV image segmentation strategies, we propose a method that is closely related to flight planning. The number of image blocks derived from a single image varies according to the heading and lateral overlap. The evaluation of different block extraction scenarios (4, 9, and 40 blocks) showed that objects close to the image center have minimal distortion and the highest recognition accuracy. The efficiency of single image analysis suggests a linear relationship between the number of image blocks and the recognition efficiency.

Limitations of this study include the complex nature of maize hybridization field scenarios and the strong influence of sunlight on recognition accuracy. The applicability of the model, based on the current datasets constructed from visually optimal images, may be limited for different scenarios. Although data augmentation considered the effects of high light and shadows, it did not comprehensively address the underlying problem. Future work will focus on evaluating more highlighted/shadowed images to enrich the datasets and improve model generalizability. Furthermore, a model with consistently high detection accuracy across all tassel development stages remains elusive. The current recommendation is to use a combination of several models for inference, but this significantly increases the computational cost and time. Future efforts will be devoted to refining the data annotation and model evaluation methods to develop a more efficient tassel detection model.

### Conclusion

This study developed a technical scheme for assessing tassel states in maize hybridization fields. This was achieved through a series of optimizations in the annotation methods, identification and selection of detection models, and block pattern evaluations, leading to effective detections of the tassel states. A valuable solution for annotating dynamic developmental objects such as tassels was proposed, and its effectiveness was evaluated. For tassel detection model selection, a comprehensive model performance evaluation method was provided; it selects the best model that is faithful to the currently available data. In terms of the efficiency and accuracy of the tassel state assessment, a quantitative evaluation was carried out using UAV image block patterns, which ultimately produced direct analysis results based on drone images, and the rapid identification of the tassel states. This study, which is tailored to real-world application scenarios, provides new insights into the analysis of tassel states in maize hybridization fields using drones. As a result, a novel intelligent system based on UAV remote sensing imagery can be developed in the future for rapid, large-scale semantic analysis of maize hybridization fields, which will reduce the reliance on manual field surveys and assist managers in their decision-making.

## Data Availability

The data used to support the findings of this study are available upon request from the corresponding author.
